# Depletion of Cellular Pre-Replication Complex Factors Results in Increased Human Cytomegalovirus DNA Replication

**DOI:** 10.1371/journal.pone.0036057

**Published:** 2012-05-07

**Authors:** Tamara Evans Braun, Emma Poole, John Sinclair

**Affiliations:** Department of Medicine, University of Cambridge, Addenbrooke’s Hospital, Cambridge, United Kingdom; Karolinska Institutet, Sweden

## Abstract

Although HCMV encodes many genes required for the replication of its DNA genome, no HCMV-encoded orthologue of the origin binding protein, which has been identified in other herpesviruses, has been identified. This has led to speculation that HCMV may use other viral proteins or possibly cellular factors for the initiation of DNA synthesis. It is also unclear whether cellular replication factors are required for efficient replication of viral DNA during or after viral replication origin recognition. Consequently, we have asked whether cellular pre-replication (pre-RC) factors that are either initially associated with cellular origin of replication (e.g. ORC2), those which recruit other replication factors (e.g. Cdt1 or Cdc6) or those which are subsequently recruited (e.g. MCMs) play any role in the HCMV DNA replication. We show that whilst RNAi-mediated knock-down of these factors in the cell affects cellular DNA replication, as predicted, it results in concomitant increases in viral DNA replication. These data show that cellular factors which initiate cellular DNA synthesis are not required for the initiation of replication of viral DNA and suggest that inhibition of cellular DNA synthesis, in itself, fosters conditions which are conducive to viral DNA replication.

## Introduction

Replication from the single origin of lytic replication (oriLyt) of HCMV requires the proteins from 11 loci of the HCMV genome, 6 core replication proteins and 5 transacting or cell survival factors in *ori*Lyt amplification assays [1]. The core viral replication proteins are a single stranded binding protein (UL57); the helicase-primase complex (UL70, UL102 and UL105) [2]; the viral DNA polymerase (UL54) and the polymerase accessory factor (UL44) [3] [4]. UL44, essential for virus DNA replication [5], [6], localises to viral replication compartments in infected cells [7] and been shown to interact with a potential replication initiator UL84 [8] HCMV does not encode a functional origin binding protein (OBP) and, whilst it has been suggested that this role is carried out by UL84 and IE86 [9], whether this process involves cellular DNA replication proteins is not known.

The control of cellular and viral DNA replication during infection is intimately linked. Upon infection, HCMV pushes cells through the G1/S check point into early S phase and it appears that one mechanism for this is via IE86 activation of E2F [10] [11] [12] [13]. Once in S phase, cells arrest and do not replicate their DNA. Both advance into S phase and then cell cycle arrest have been attributed to IE86 [14] [15] [16] [17].

It has been suggested that the inhibition of cellular DNA replication by HCMV is due to disruption of the assembly of the cellular pre-replication complex (pre-RC) [18] [13] which constitutes the cellular origin binding complex. The key components of the pre-RC are the origin recognition complex (ORC) [19], [20] [21] [22], [23], [24] [25] Cdc6 [24], [25], Cdt1 [26] [24] [27] and the minichromosome maintenance proteins (MCMs) [28] [29] [30].

Several studies have addressed the expression and loading of pre-RC proteins onto cellular DNA during HCMV infection [18] [13] [31]. Except for a decrease in Cdt1 and an increase in Cdc6, HCMV infection of quiescent fibroblasts results in little change in the levels of expression of pre-RC proteins [18]. Similarly, analysis of loading of pre-RCs onto cellular DNA also showed that HCMV infection had little effect on chromatin loading of Cdt1 and the ORCs [13]. In contrast, loading of MCMs onto cellular chromatin appears to be prevented during HCMV infection [13] and this has been shown to be due, at least in part, to the viral protein UL117 [31].

In this study, we have used siRNA knockdowns of Cdc6, Cdt1, ORC2 and MCM7 to address their role, if any, in HCMV DNA replication and show that infection of cells depleted for these proteins results in enhanced viral lytic DNA replication. These data formally rule out a requirement for cellular pre-RC proteins for HCMV DNA replication and underscores the view that that inhibition of cellular DNA synthesis, per se, is important for efficient HCMV DNA replication.

**Table 1:** Primer sequences and PCR conditions.

Primer Target	Sense Primer Sequence	Anti-sense Primer Sequence	Annealing Temp.(°C)	Mg Conc.(mM)	Product size(Kb)
IE	CGTCCTTGACACGATGGAGT	ATTCTTCGGCCAACTCTGGA	55	2.5	194
pp28	GAGGATGACGATAACGAGGA	TCAAACAGCACATTAGACACACGG	55	2	548
MIEP	TGGGACTTTCCTACTTGG	CCAGGCGATCTGACGGTT	50	1.5	285
GAPDH	GAGTCAACGGATTTGGTCGT	TTGATTTTGGAGGGATCTCG	60	2.5	236
MIEP (QPCR)	CCAAGTCTCCACCCCATTGAC	GACATTTTGGAAAGTCCCGTTG	60	4.5	71
GAPDH (QPCR)	CGGCTACTAGCGGTTTTACG	AAGAAGATGCGGCTGACTGT	60	5.5	189

## Methods

### Cell Culture and Virus Infection

Human foetal foreskin fibroblasts (HFFFs) were obtained from the American Type Culture Collection. Cells were grown in Eagle’s minimal essential medium (Invitrogen) supplemented with 2 mM L-glutamine, 10% fetal calf serum (FCS), 100 U/ml penicillin and 100 U/ml streptomycin (EMEM-10). Cells were grown at 37°C in 5% CO_2_. For synchronisation of cells in G0 phase of cell cycle, cells were serum starved for 24 hours using EMEM without added FCS (EMEM Wash). To induce the cells to enter S phase, EMEM washed was replaced with EMEM-10.

HCMV Toledo strain [32] was used for all experiments. Virus was cultured in HFFFs and the titre was determined by TCID50. For replication assays, cells were infected at an MOI of 1.

### siRNA Transfection

All siRNAs were provided by Dharmacon and were made up in 1x Dharmacon siRNA buffer to a concentration of 20 µM. siRNAs were stored as aliquots at -80°C to avoid multiple freeze thaw cycles. Sequences were as follows:

Scramble control (Sc) (custom) GCGCGCUUUGUAGGAUUCG. Cdc6 (sigenome) GAGCAGAGAUGUCCACUGAUU. Cdc6 (custom) ACUAGAACCAACAAAUGUC. Cdt1 (sigenome) CCAAGGAGGCACAGAAGCAUU. Cdt1 (custom) GAUAAAGAAAUCCACCCCG. Cdt1 (Higa et al., 2003) GUACCCCCGAGGCCCCAGA. ORC2 (sigenome) GAAGAAACCUCCUAUGAGAUU. ORC2 (custom) GGAGGAGCUAAAUUGAAGA. MCM7 (sigenome) GGAAAUAUCCCUCGUAGUAUU. UL44 1 (custom) GCCGUACAAGACGGCUAU. UL44 2 (custom) UUACUUCAAGACGCGAAA.

Transfection of siRNAs was carried out as previously described [33]. In detail, where two or three different siRNAs were available for one target, equivalent amounts of siRNAs were pooled. For transfection of HFFF cells on a six well plate, cells were grown to 60% confluency and EMEM-10 was replaced with Opti-MEM (Invitrogen) four hours prior to transfection. 192 pmol siRNA was added to 470.4 µl Opti-MEM and incubated for 5 minutes at room temperature, this was then added to a mix of 4.8 µl Lipofectamine-2000 (Invitrogen) and 475.2 µl Opti-MEM, which had been freshly made and incubated for 5 minutes at room temperature. The siRNA-Lipofectamine solutions were incubated for 20 minutes at room temp. 960 µl of the each solution was then added to each well of plated cells and mixed gently. EMEM-10 or serum-free EMEM where indicated, was replaced 3–6 hours post-transfection. The protocol was scaled down for HFFFs on 8 well slides so that 20 pmol siRNA was used per well. ‘Mock transfection’ refers to cells treated with Lipofectamine without the addition of siRNA. Specifically in Figure S2, siRNA-treated cells were maintained in EMEM wash for 24 h prior to infection.

### Western Blot Analysis

Cells and media were harvested 24 and 96 hours post-infection using a cell scraper. Cell pellets were re-suspended in Laemmli sample buffer (0.125 M Tris-HCl pH 6.8, 25% v/v glycerol, 1.25% v/v 2-Mercaptoethanol, and 1.25% SDS in distilled water), boiled for 5 minutes and centrifuged at low speed for 2 minutes. Proteins were separated by SDS PAGE and transferred onto Hybond C nitrocellulose filters (Amersham). Following transfer, the membrane was incubated in blocking solution (5% dried skimmed milk, 0.1% Tween20 in PBS) for 30 minutes at room temperature. The membrane was then incubated with the primary antibody (diluted in blocking solution) for 1 hour at room temperature or overnight at 4°C. Following this, the filter was washed (0.1% Tween20 in PBS). The filters were then incubated for 20 minutes at RT with the secondary antibody (diluted in blocking solution). Antibody binding was detected using the ECL or ECL+ system from Amersham (as described by the manufacturers’ instructions) and X-ray film. Exposure times varied from 1 second to 1 hour.

Antibodies: Anti-pp28 (mouse, Virusys) used 1 in 500, Anti-beta actin (goat, Abcam) used 1 in 1000, Anti-UL44/p52/ICP36 (mouse, Autogenbioclear) used 1 in 1000, Anti-gapdh HRP (rabbit, Abcam) used 1 in 1000, Anti-MCM7 (goat, Abcam) used 1 in 1000, Anti-ORC2 (mouse, Stressgen) used 1 in 1000, Anti-mouse HRP (donkey, Dako) used 1 in 2000, Anti-goat HRP (donkey, Dako) used 1 in 2000.

### RNA Extraction

1×10^6^ Cells were lysed in 1 ml TRI REAGENT and RNA was extracted according to manufacturers instructions. RNA was treated with DNase (Promega) prior to reverse transcription according to manufactueres instructions. RNA was reverse transcribed using the Promega reverse transcription kit according to the manufactures instruction. cDNA was stored at −20°C and analysed by PCR.

### Preparation of DNA from Cells

Cells and media were harvested using a cell scraper and pellets were washed twice in 50 mM EDTA. The pellets were then re-suspended in 0.25 ml TE. 0.25 ml PK buffer (300 mM NaCl, 200 mM tris pH 7.5, 25 mM EDTA) was added followed by 50 µl 10% SDS to lyse cells. Samples were then incubated at 37°C for 1–1.5 hours with 50 µl RNase A (2 mg/ml) to remove the RNA. Protein was degraded by the addition of 10 µl proteinase K (20 mg/ml) and incubation for a further 2 hours. DNA was then extracted by phenol chloroform extraction and ethanol precipitation in the presence of sodium acetate. Finally, the pellets were re-suspended in 100 µl TE.

### Standard Polymerase Chain Reaction (PCR)

PCR reactions for replication analysis were carried out using Bioline Red PCR mix in accordance with the manufacturer’s protocol. The PCR conditions used were denaturation at 94°C for 40 seconds, annealing for 40 seconds (temperature was Tm dependent) and extension at 72°C for 90 seconds. PCR primer sequences are shown in table 1. PCR for Cdc6 was carried out as previously published [34]. DNA products were analysed by separation on a 1–2% agarose gel at 100V (containing ethidium bromide) and visualised under UV.

### Quantitative PCR (QPCR)

QPCR was carried out using the taqman system (primers shown in table 1). Mixes were made with Buffer, MgCl_2_ and Taq polymerase (Qiagen), dNTPs (Bioline), primers (Sigma) and probes (TIB MolBiol). GAPDH probe: Cy5-CACGTAGCTCAGGCCTCAAGACCT-BBQ. MIEP probe: AFAM-TGGGAGTTTGTTTTGGCACCAAA-TMR.

For analysis of levels of viral DNA, QPCR to the MIEP region of the HCMV genome was performed. QPCR reactions were set up as follows: 2 µl 10x Hifidelity Polymerase buffer, 2.4 µl MgCl_2_ (25 mM), 1.6 µl dNTP mix (25 mM each DNTP) 0.6 µl AD169 forward primer (10 µM), 0.1 µl AD169 reverse primer (10 µM), 0.05 µl AD169 probe (10 µM), 0.16 µl HotStar HiFidelity DNA polymerase, 5.09 µl water and 8 µl DNA sample.

Results were normalized against cellular DNA content measured using a QPCR of the GAPDH gene. Reactions were set up as follows: 2 µl 10x HiFidelity Polymerase buffer, 3.2 µl MgCl_2_ (25 mM), 1.6 µl dNTP mix (Bioline, 25 mM each DNTP) 0.6 µl huGAP forward primer (10 µM), 1.8 µl huGAP reverse primer (10 µM), 0.1 µl huGAP probe (10 µM), 0.16 µl HotStar HiFidelity DNA polymerse, 2.54 µl water and 8 µl DNA sample.

Samples were run in a RotorGene 3000 thermocycler alongside reactions containing known concentration of target DNA. These were used to produce a standard curve and to allow quantitative analysis of the sample DNA. The reaction conditions were as follows: an initial denaturation and activation of enzyme at 95°C for 15 min, then 45 cycles of annealing/extension at 60°C for 60 sec then denaturation at 95°C for 15 sec.

### BrdU Staining

After treatment with siRNAs, HFFFs on 8 well slides were cultured in EMEM-wash for 72 h. At this time, 10 nM Bromodeoxyuridine (BrdU) in EMEM-10 was added for 4 hours at 37°C. After this period, slides were fixed in 1% paraformaldehyde for 10 minutes then washed in PBS 3 times for 5 minutes each. Slides were then permeabilized by incubation with 1% triton X and washed 3 times for 5 minutes each. To allow detection of BrdU by the antibody, the DNA was then denatured by incubation with 4 M HCL for 10 minutes after which the slides were washed 4 times for 5 minutes. 50 µl neat Rat anti-BrdU supernatant (Abd Serotec, OBT00305) was added to each well and incubated for 1 hour at room temperature. Following this, the slides were washed 3 times for 5 minutes with PBS. Secondary anti-rat alexaflour 594 (Molecular Probes), 1 in 100 dilution, was added to each well and left for 1 hour. Slides were washed as before and were then left to dry, mounted and viewed by fluorescence microscopy. Images were analysed using Image pro software.

### Propidium Iodide Staining

After treatment with siRNAs, cells were cultured in EMEM-10 for 48 h. 1×10^6^ cells were washed twice in PBS then harvested using cell dissociation buffer before being centrifuged at 1800 rpm for 5 min in a MSE Falcon 6/300 centrifuge. Cell pellets were re-suspended in 1 ml 70% ethanol and left for a minimum of 5 minutes at -20°C. The fixed cells were then washed in PBS and re-suspended in 1 ml PBS plus 10 µl RNAse (2 mg/ml). Samples were left for 30 minutes at room temperature and then spun at 2500 rpm as before. The pellet was then re-suspended in 1 ml propidium iodide (50 mg/ml) and incubated for 1 hour at room temperature. Finally, cells were washed twice in PBS, re-suspended in 0.5 ml PBS and analysed by flow cytometry. Single cells were gated and percentages of cells in the G0/G1 phases of the cell cycle were calculated using winMDi.

## Results

### Removal of Cellular ORC2 Results in Increased HCMV DNA Replication

**Figure 1:**
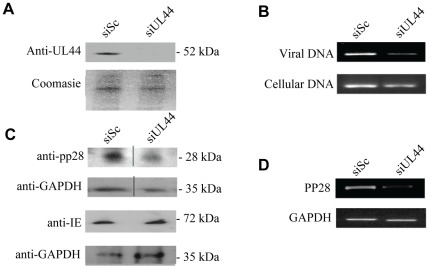
Knockdown of UL44 results in a decrease in viral DNA replication. HFFFs transfected with siUL44 or scramble siRNA were infected at 48 hours post-transfection with HCMV Toledo strain at an M.O.I. of 1. 96 hours post infection, knockdown of UL44 by siRNA was confirmed by western blot analysis (A). Levels of viral DNA were also assessed by PCR (B). PP28 (at 96 h post-infection) and IE (at 24 h post-infection) was assayed by western blot (C). Similarly, pp28 and IE RNA was also assessed at 96 h post-infection by RT-PCR (D).

In order to determine the effect of removal of cellular DNA replication factors on HCMV infection, we used RNAi technology to deplete expression of the cellular proteins using transient delivery of specific siRNAs; a protocol which has been used effectively on a number of HCMV genes to specifically inhibit viral gene expression [33] [35]. Initially, as a proof of principle, we tested the effect of siRNA knock down of HCMV UL44, an essential viral DNA polymerase accessory protein [5], on viral DNA replication. Figure 1A shows that UL44 siRNAs specific for UL44 effectively prevent the expression of UL44 protein by western blot as late as 96 h post-infection. Consistent with the known essential function of UL44 as a viral DNA polymerase accessory protein [36], analysis of accumulation of viral DNA at 96 h post-infection showed that levels of viral DNA were also substantially reduced in UL44-specific siRNA treated cells (figure 1B). We also noted that siRNAs to UL44 prevented accumulation of the late protein pp28 RNA or protein (figure 1C and D), whose expression is dependent on viral DNA replication [37], but did not prevent expression of the major IE72 protein at 24 h post-infection (Figure 1C and statistical analysis shown in Figure S1A). Again, this was consistent with the view that siRNAs to UL44 specifically inhibited viral replication but not virus entry or immediate early events. Similar results have observed by others using anti-sense RNAs to UL44 in immortalised glioblastoma cells [36].

**Figure 2:**
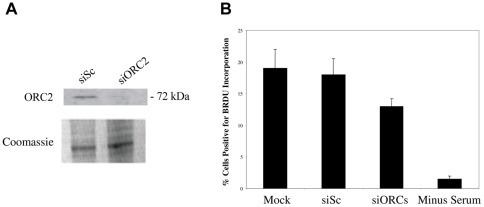
ORC2 knock-down decreases cellular DNA synthesis. Cells were mock transfected, transfected with control siRNA or transfected with a siRNA to ORC2 in the presence of serum. 48 hours post-transfection, cells and media were harvested by trypsinisation and centrifugation and analysed by western blot for ORC2 (A). Additionally, mock treated cells or cells transfected with siRNAs were maintained for 72 hours post-transfection in EMEM-wash and then BrdU was added to the cell media in the presence of serum for 4 hours after which cells were fixed and stained for BrdU incorporation and samples were analysed by immunofluorescence. 5 independent fields of view were counted for BrdU staining and the percentage of and the percentage of BrdU positive cells was calculated. Error bars are mean plus standard error margin (B). Numbers of BrdU positive cells are also shown for cells continually deprived of serum (minus serum) (B).

**Figure 3:**
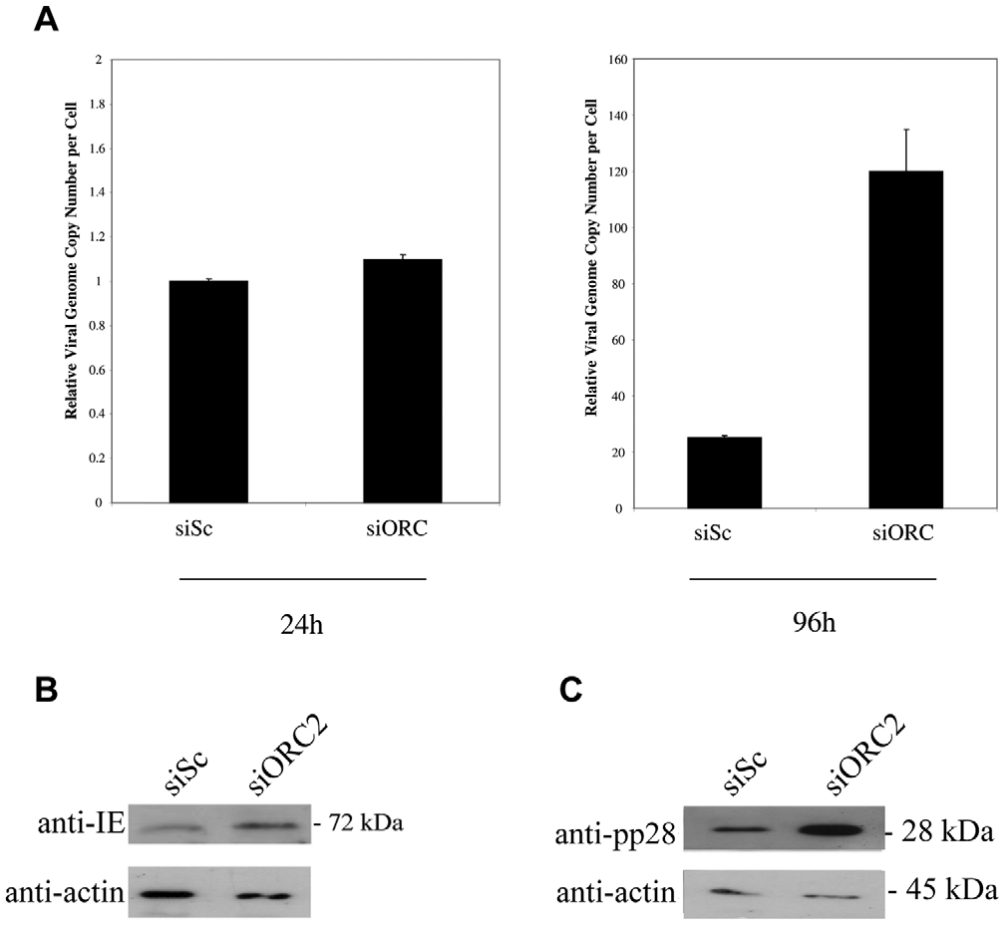
Knockdown of ORC2 increases viral DNA replication. HFFFs were transfected with siRNAs ORC2 or a scramble control in the presence of serum. 48 hours post-transfection cells were infected at an MOI of 1. Cells and media were harvested at 24 (A and B) and 96 (A and C) hours post-infection and DNA or protein isolated. Levels of viral DNA were quantified by qPCR and normalized against cellular DNA (A). Error bars shown are mean plus standard error margin. Alternatively, cells were analysed at 24 h post-infection for the viral gene product IE (B) or at 96 h post-infection for the viral gene product pp28 (C) by western blot.

On the basis that siRNA delivery, targeting a factor known to be essential for viral DNA synthesis did, indeed, specifically inhibit viral DNA replication, we next tested the effect of knock-down of a cellular factor normally constitutively associated with cellular origins of replication. We initially chose to target cellular ORC2 which is a key component of the origin recognition complex and acts as a platform for the assembly of the pre-RC [19], [20] [21] [22] [23]. Delivery of siRNAs specific for ORC2 resulted in good levels of ORC2 depletion (figure 2A) which also resulted in a decrease in cellular DNA synthesis (figure 2B). The effect of ORC2 depletion on HCMV infection was profound. We observed a substantial increase in the level of viral DNA replication at 96 h post-infection in siORC2 treated cells compared to cells treated with control siRNA (figure 3A). This could not be attributed to increased uptake of viral DNA in siORC2 treated cells as evidenced from the equivalent levels of viral DNA in control siRNA treated cells and siORC2 treated cells at 24 h post-infection (figure 3A). Removal of ORC2 also led to increased levels of IE72 expression at 24 h post-infection (figure 3B and statistical analysis shown in figure S1A) and, consistent with the increase in viral DNA replication, an increase in expression of the true late protein pp28 (figure 3C).

Taken together, these data show in the absence of ORC2, an initiating factor for the cellular pre-RC, viral IE gene expression and subsequent viral DNA replication is substantially enhanced.

### Removal of Cellular Cdt1 or Cdc6 Causes an Upregulation of HCMV DNA Replication

Clearly, removal of the cellular factor ORC2 during HCMV infection did not impede viral DNA synthesis and actually led to an increase in viral replication. Consequently, we next asked whether removal of cellular proteins such as Cdt1 and Cdc6, which are responsible for licensing cellular replication origins by recruiting MCM proteins [30] [19] [24] [25] [26] [27], had any effect on infection. It is interesting to note that at IE/early times of HCMV infection these factors are differentially regulated: Cdt1 is downregulated whereas Cdc6 is upregulated [18]. Such an increase in Cdc6, could reflect a requirement for this cellular factor during infection. Similarly, in our laboratory, we have identified an interaction between the viral gene IE86 and Cdt1 (Bain *et al.,* manuscript in preparation). The role for this interaction between Cdt1 and IE86 is not known. However, one possibility is that the interaction between Cdt1 and IE86, which itself binds close to the viral origin of replication, results in recruitment of Cdt1 to viral origins of replication, perhaps facilitating viral DNA replication. Alternatively, given that steady state levels of Cdt1 are reduced during HCMV infection [18], IE86 may sequester Cdt1 to prevent functions of Cdt1 which are perhaps detrimental to viral replication.

**Figure 4:**
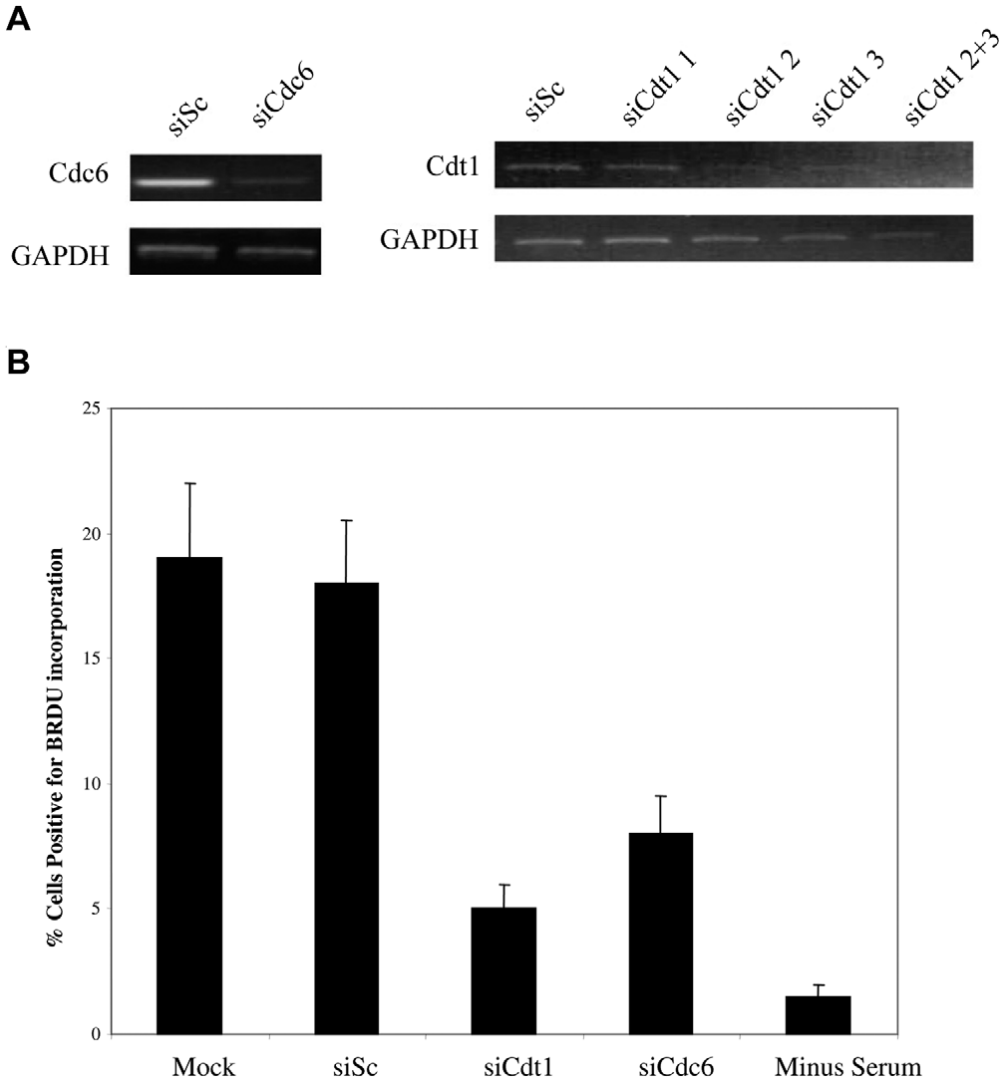
Cdt1 and Cdc6 knock-down reduces cellular DNA synthesis, as expected. Cells were transfected with control siRNA or transfected with a siRNA to Cdt1 or Cdc6 in the presence of serum. 48 hours post-transfection cells and media were harvested by trypsinisation and centrifugation. Cells were analysed by RT-PCR for Cdt1 or Cdc6 mRNA levels (A). Additionally, mock treated cells or cells transfected with siRNAs cells were maintained for 72 hours post-transfection in EMEM-wash and then BrdU was added to the cell media for 4 hours after which cells were fixed and stained for BrdU incorporation and samples were analysed by immunofluorescence. 5 independent fields of view were counted for BrdU staining and the percentage of BrdU positive cells was calculated (B). Error bars are mean plus standard error margin. Numbers of BrdU positive cells are also shown for cells deprived of serum (minus serum) (B).

To address this, we first ensured that we were able to remove Cdt1 or Cdc6 by RNAi and confirmed the effects of their removal on cellular DNA synthesis. Figure 4A clearly shows that Cdt1 and Cdc6 mRNA levels were decreased following specific siRNA treatment. Figure 4B also shows that removal of either Cdt1 or Cdc6 led to a considerable decrease in the ability of cells to synthesis cellular DNA.

**Figure 5:**
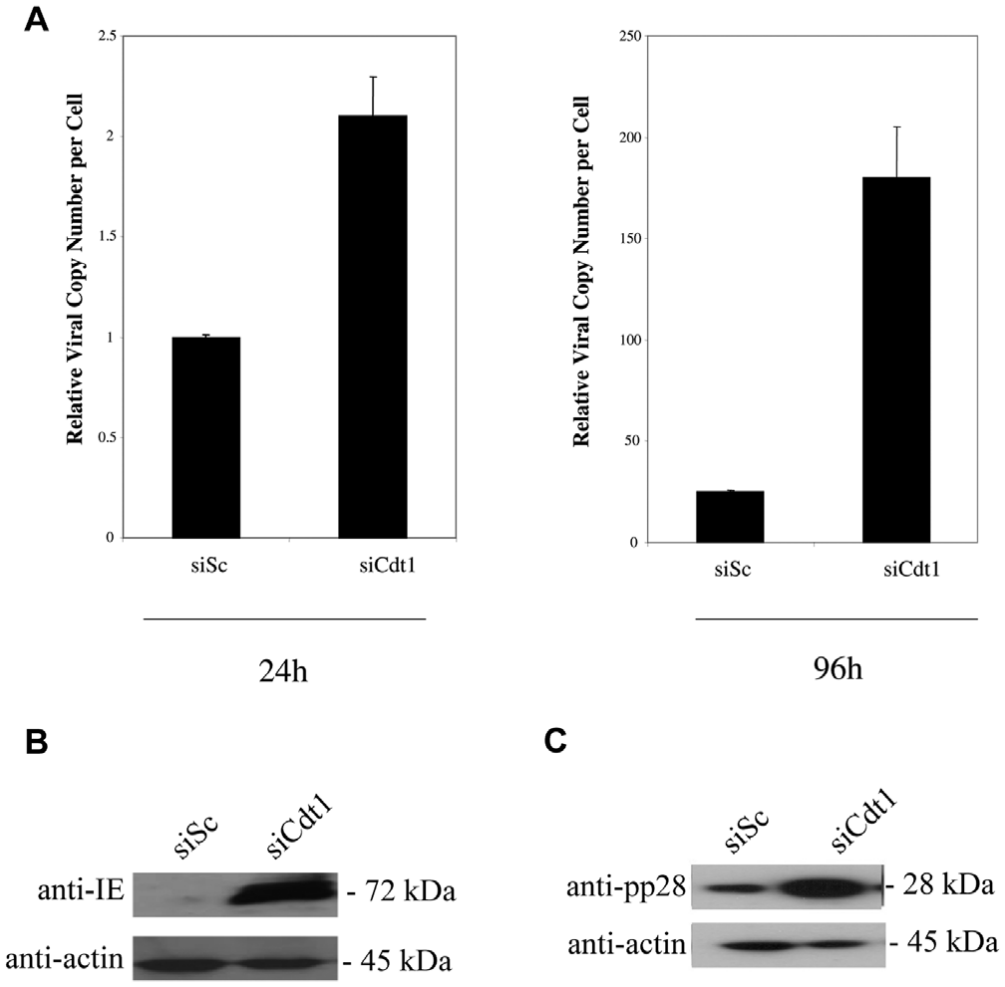
Knockdown of Cdt1 increases viral DNA replication. HFFFs were transfected with siRNAs to Cdt1or a scramble control in the presence of serum. 48 hours post-transfection cells were infected at an MOI of 1. Cells and media were harvested at 24 and 96 hours post-infection and DNA isolated. Levels of viral DNA were quantified by qPCR and normalized against cellular DNA (A). Error bars shown are mean plus standard error margin. Alternatively, cells were also analysed at 24 h post-infection for the viral gene product IE (B) or at 96 h post-infection for the viral gene product pp28 (C) by western blot.

In contrast, removal of Cdt1 during the course of a virus infection had profound effects on viral DNA replication. Figure 5A shows that treatment of cells with siRNAs specific for Cdt1 resulted in a major increase in virus genomes at 96 post-infection compared to cells treated with scrambled siRNA, although we note that knock-down of Cdt1 resulted in a small increase in delivery of viral genomes (about 2 fold, see figure 5A). Interestingly, removal of Cdt1 during infection also led to a profound increase in levels of IE72 expression at 24 h post-infection (figure 5B and statistical analysis shown in figure S1A) and, consistent with increased levels of viral DNA replication, also resulted in increased levels of the true late pp28 protein at 96 h post-infection (figure 5C).

**Figure 6:**
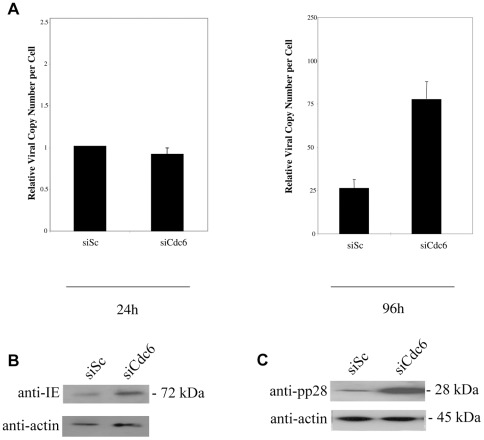
Knockdown of Cdc6 increases viral DNA replication. HFFFs were transfected with siRNA Cdc6 or a scramble control in the presence of serum. 48 hours post-transfection cells were infected at an MOI of 1. Cells and media were harvested at 24 and 96 hours post-infection and DNA isolated. Levels of viral DNA were quantified by qPCR and normalized against cellular DNA. Error bars shown are mean plus standard error margin (A). Alternatively, cells were also analysed at 24 h post-infection for the viral gene product IE (B) or at 96 h post-infection for the viral gene product pp28 (C) by western blot.

Similar to removal of Cdt1, removal of Cdc6 during infection (figure 6) also resulted in increased viral DNA replication (figure 6A) with a concomitant increase in IE72 expression at 24 h post-infection (figure 6B and statistical analysis shown in figure S1A) and, consistent with an increase in viral DNA replication, increased pp28 expression at 96 h post-infection (figure 6C). However, the level of increase in IE gene expression, viral DNA replication and pp28 expression was not as high as that induced by removal of Cdt1.

### Removal of Cellular MCM7 Causes an upregulation of HCMV DNA Replication

**Figure 7:**
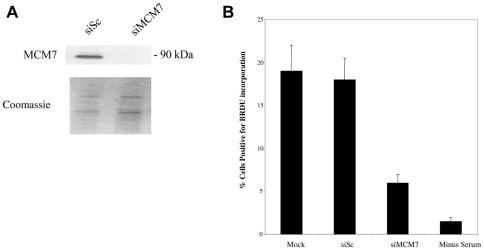
MCM7 knock-down reduces cellular DNA synthesis, as expected. Cells were transfected with control siRNA, or transfected with a siRNA to MCM7. 48 hours post-transfection cells and media were harvested by trypsinisation and centrifugation. Cells were analysed by western blot for MCM7 content (A). Additionally, mock treated cells or cells transfected with siRNAs were maintained for 72 hours post-transfection in EMEM-wash and then BrdU was added to the cell media for 4 hours after which cells were fixed and stained for BrdU incorporation and samples were analysed by immunofluorescence. 5 independent fields of view were counted for BrdU staining and the percentage of BrdU positive cells was calculated. Error bars are mean plus standard error margin (B). Numbers of BrdU positive cells are also shown for cells deprived of serum (minus serum) (B).

MCM7 is an integral component of the MCM complex which is essential for both initiation and elongation of DNA replication and is recruited as part of the pre-RC during cellular DNA replication [29] [30] [28]. An integral function of the MCM complex is helicase activity and once MCM complex has been recruited to the cellular origin of replication a fully licensed replication origin exists. Although HCMV encodes its own replicative helicase-primase complex in the viral UL70, UL102, and UL105 gene products [38] [39], it is not known if cellular MCMs are required for viral DNA replication. Consequently, we also analysed HCMV DNA replication in cells in which MCM7 had been depleted by RNAi. To test whether MCM7 is required for the replication of viral DNA, siRNA molecules were designed to target MCM7. Figure 7A shows that treatment of cells with MCM7 specific siRNAs led to a good reduction in steady state levels of MCM7 protein by western blot analysis. Consistent with the known effects of MCM7 depletion [40], this also led to a profound inhibition of cellular DNA synthesis (figure 7B).

**Figure 8:**
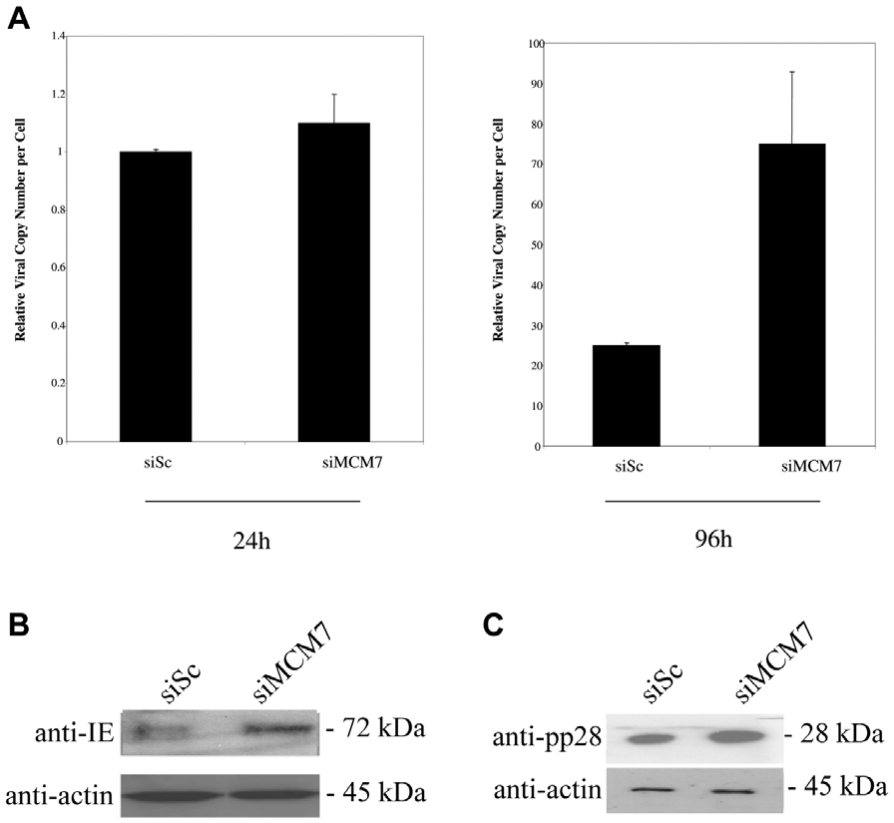
Knockdown of MCM7 increases viral DNA replication. HFFFs were transfected with siRNAs to MCM7 or a scramble control in the presence of serum. 48 hours post-transfection cells were infected at an MOI of 1. Cells and media were harvested at 24 and 96 hours post-infection and DNA isolated. Levels of viral DNA were quantified by qPCR and normalized against cellular DNA. Error bars shown are mean plus standard error margin (A). Alternatively, at 24 h post-infection cells were analysed for the viral gene product IE (B) or at 96 h post-infection for the viral gene product pp28 (C) by western blot.

Nevertheless, this RNAi-mediated reduction in MCM7 during infection with HCMV once again led to a robust increase in cellular DNA synthesis at 96 h post-infection (figure 8A) which was not due to differences in input genome and also led to concomitant increases in IE72 expression at 24 h post-infection (figure 8B and statistical analysis shown in figure S1A) and pp28 expression at 96 h post-infection (figure 8C).

In the experiments so far, knock-downs of specific pre-RC components were carried out in a background of serum which would otherwise provide the necessary cellular conditions required for cellular DNA replication. We, therefore, asked whether the effects of knock-down of pre-RC components in serum-depleted conditions would also result in similar effects on viral IE gene expression. Intriguingly, knock-down of pre-RC components in serum-free medium showed only marginal effects on viral IE gene expression (see figure S2). One interpretation of this is that under conditions where there is no competition between the virus and the cell for cellular factors required for DNA replication, because cellular DNA replication is already potently inhibited by serum-deprivation, additional knock-down of specific pre-RC components have little additional effects.

Taken together, the data suggest that knock-down of ORC2, Cdt1 and Cdc6 or MCM7 in a background of conditions otherwise conducive to cell proliferation, leads to an enhancement of viral DNA replication.

## Discussion

The pre-RC represents a key component of the cellular replication machinery. The complex assembles from its components in a stepwise manner onto cellular DNA replication origin. This culminates in phosphorylation of the MCMs which leads to origin melting and recruitment of the DNA polymerase [41]. Cellular pre-RC proteins have been shown to be involved in herpesvirus latent replication. Both MCM and ORC proteins have been shown by ChIP assays to bind EBV oriP [42] [43] where they appear to be recruited to the origin by EBNA1 [44]. ORC and MCMs have also been shown to play a role at the latent origin of KSHV [45] [46] [47]. However, to date, it is not known if such cellular factors are required for herpesvirus lytic replication.

In, for example, herpes simplex virus (HSV) lytic replication, viral DNA synthesis is initiated by an origin binding protein, UL9. HCMV does not encode a de facto origin binding protein, so the exact mechanism by which it initiates viral lytic DNA replication is unclear. Although, recently, it has been shown that HCMV UL84 can interact with the viral origin of replication and may function as a replication initiator via interactions with C/EBP [38], [48] [49], other work has shown that UL84 is dispensible for HCMV lytic replication [50]. Consequently, we have analysed whether initiation of HCMV DNA replication involves host cell pre-RC factors.

We tested whether viral DNA replication required factors involved in three defined stages of cellular pre-RC formation; these were an initiation factor (ORC2), two recruiting factors (Cdt1 and Cdc6) and a recruited factor (MCM7).

Our data clearly showed that none of the components of the cellular pre-RC were required for viral DNA replication. On the contrary, removal of cellular components required for any stage of cellular pre-RC formation, resulting in inhibition of cellular DNA replication, actually led to robust increases in viral DNA replication. It is well established that HCMV infection results in cell cycle arrest, suggesting that cellular DNA replication may compete against viral DNA replication [10] [11] [12] [51] [17] [52] [53].

Thus, compromising cellular DNA replication by removing cellular components required for cellular DNA synthesis likely results in a cellular environment which is more conducive to viral DNA replication.

We also noted that knock-down of ORC2, Cdt1, Cdc6 or MCM7 as well as resulting in increased viral DNA replication resulted in robust increases in IE72 expression at IE times of infection - this could not be explained by, for instance, an increase in delivery of viral genome to cells lacking these cellular replication proteins. It is known that HCMV only initiates IE gene expression in cells in G0/G1 or very early S phase [54], [55], [56] and it is well established that depletion of ORC2, Cdt1, Cdc6 or MCM7 can result in cell cycle arrest at G0/G1 [40] [6], [57], [58], [59], [60], [61], [62]. Consequently, we tested whether cells treated with specific siRNAs to pre-RC factors under the conditions we used for our infection assays, resulted in major differences in the number of cells in G0/G1. Delivery of siRNAs to ORC2, Cdt1, Cdc6 or MCM7 did result in low but consistent increases in cells in G0/G1 (see figure S3) which may explain, at least in part, the increased levels of IE expression in these cells. However, knock-down of Cdt1 routinely resulted in the highest increase in IE72 expression (see Figure 5B and figure S1A) and, while this may have been partly due a minor increase in genome delivery, Cdt1 knockdown did not routinely result in the highest increase in cells in G0/G1 - the highest increase in cells in G0/G1 actually resulted from treatment with siMCM7 (see figure 7 and figure S3) yet siMCM7 treated cells did not result in the highest increase in IE gene expression.

Consequently, we believe other effects besides increases in numbers of cells in G0/G1, per se, are responsible for increased efficiency of IE gene expression and viral DNA replication. These analyses are presently ongoing.

In conclusion, we have shown that the independent depletion of a number of cellular pre-RC components, which leads to arrest of cellular DNA synthesis, does not prevent HCMV DNA synthesis. On the contrary, removal of ORC2, Cdt1,Cdc6 or MCM7 led to increased viral DNA synthesis. The reasons for this are unclear, however, it is possible that arrest of cellular DNA synthesis enhances the availability of other, as yet unidentified components important for viral IE expression and viral DNA synthesis which are normally abundant during G0/G1 phase of the cell cycle.

## Supporting Information

Figure S1 **Quantification of the effects of knockdown of UL44, Cdt1, Cdc6, MCM7 or ORC2 on IE72 expression.** A) HFFFs were transfected with siRNAs as indicated or a scramble control in the presence of serum. 24 hours post-infection (at an M.O.I. of one), cells were analysed by western blot for IE expression. Data represent triplicate experiments averaged to GAPDH levels and analysed using Image J freeware.
(TIF)

Figure S2 **Cells halted at G0 by serum-starvation in the absence of Cdc6, Cdt1 or MCM7 show marginal increases in IE and pp28 expression during HCMV infection.** A) Cells transfected with siRNAs were maintained in EMEM-wash for 24 h then infected with HCMV. 24 h or 96 h post-infection, IE and pp28 protein was analysed by western blot, respectively. B) Relative levels of IE proteins in (A) were quantified using Image J freeware after normalisation to actin levels.
(TIF)

Figure S3 **Effects of knockdown of ORC2, MCM7, Cdt1, and Cdc6 on cell cycle**. HFFFs were transfected with siRNAs as labelled. After this, cells were cultured in EMEM-10 for 48 h and then stained with PI and cell cycle analysis was assayed by flow cytometry after gating for single cells. Percentages of cells in the G0/G1 phases of the cell cycle were calculated using winMDi software. Untreated cells in the absence of serum (minus serum) are also shown for comparison.
(TIF)
