# Supplementary figures and images for: Depletion of Cellular Pre-Replication Complex Factors Results in Increased Human Cytomegalovirus DNA Replication
Source: PLoS One. 2012 May 7;7(5):e36057. doi: 10.1371/journal.pone.0036057 (PMC3346814; doi:10.1371/journal.pone.0036057)

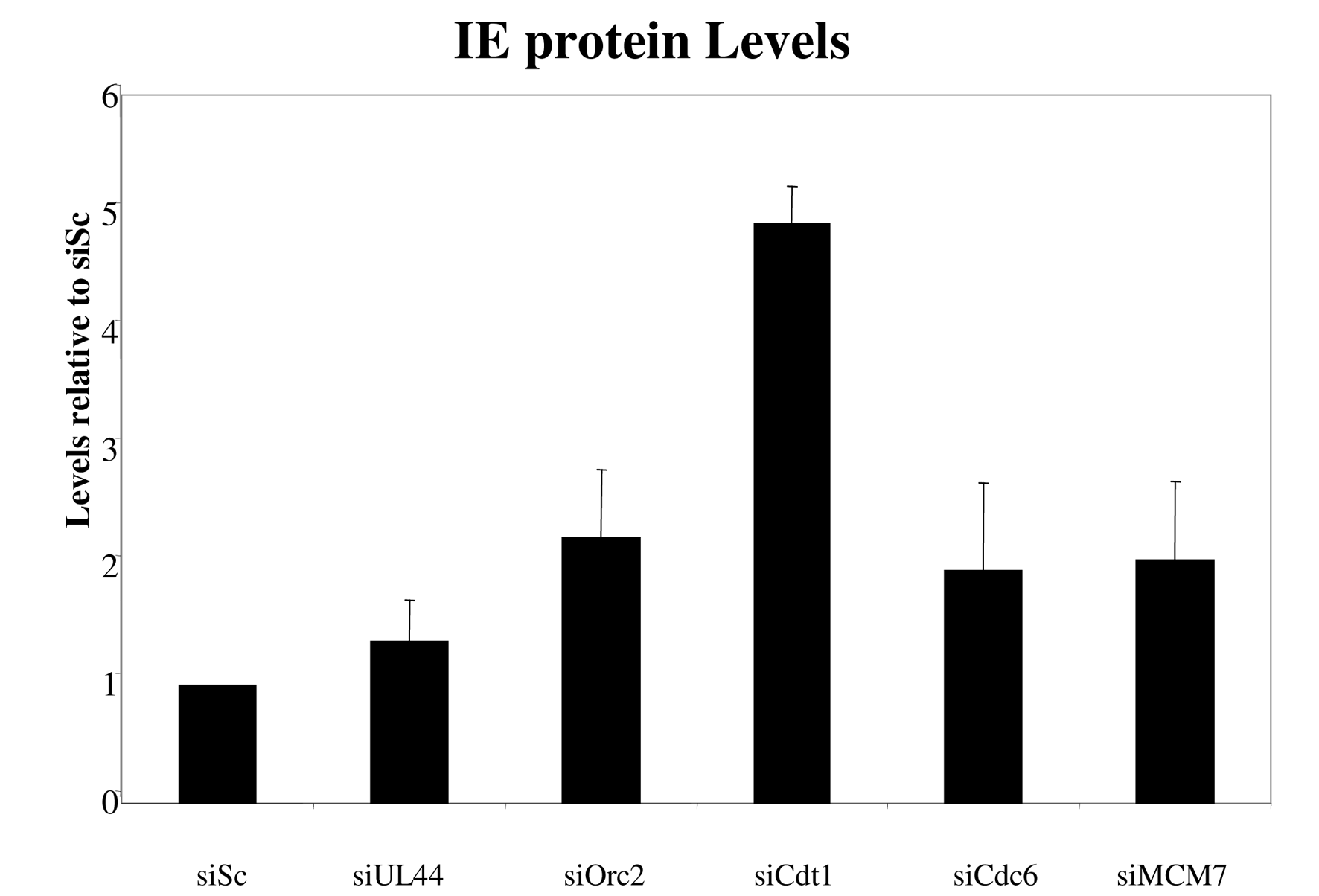

Supplement: Figure S1 — Quantification of the effects of knockdown of UL44, Cdt1, Cdc6, MCM7 or ORC2 on IE72 expression. A) HFFFs were transfected with siRNAs as indicated or a scramble control in the presence of serum. 24 hours post-infection (at an M.O.I. of one), cells were analysed by western blot for IE expression. Data represent triplicate experiments averaged to GAPDH levels and analysed using Image J freeware. (TIF) [file pone.0036057.s001.tif]

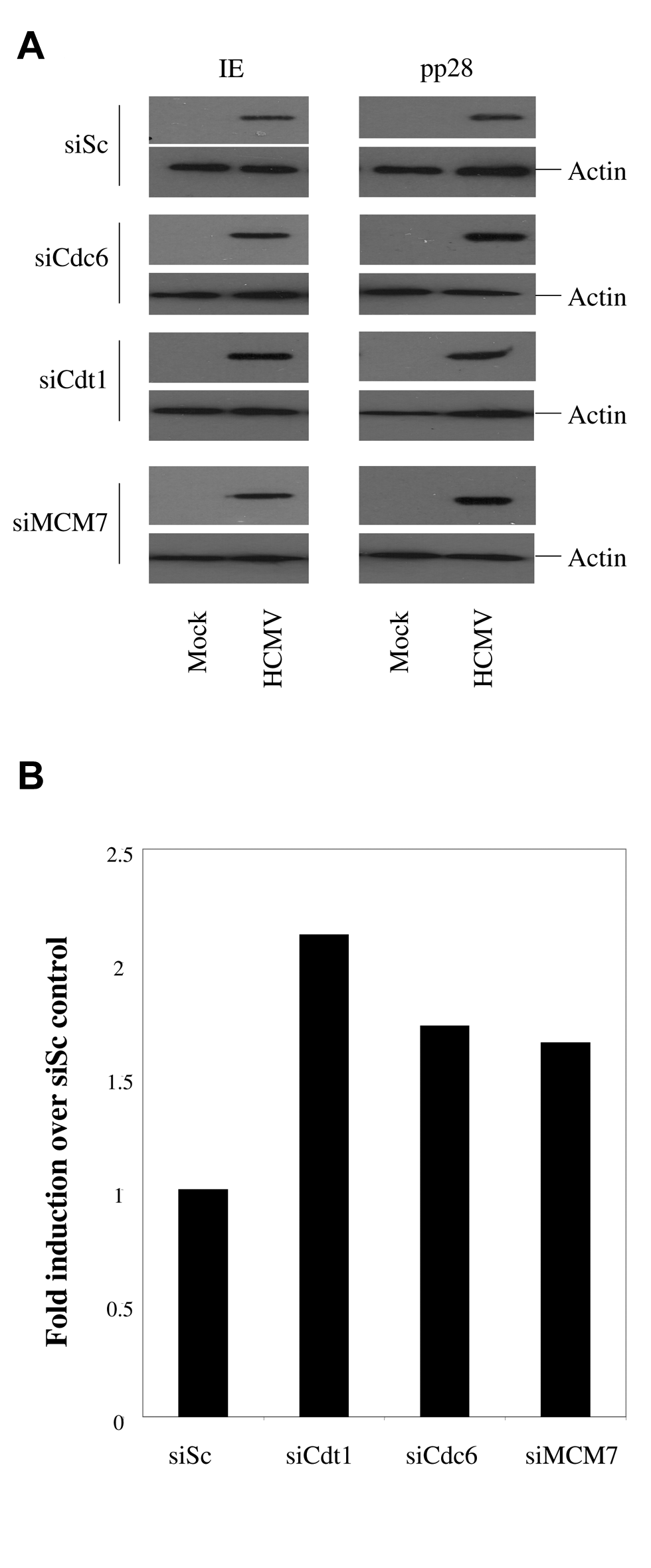

Supplement: Figure S2 — Cells halted at G0 by serum-starvation in the absence of Cdc6, Cdt1 or MCM7 show marginal increases in IE and pp28 expression during HCMV infection. A) Cells transfected with siRNAs were maintained in EMEM-wash for 24 h then infected with HCMV. 24 h or 96 h post-infection, IE and pp28 protein was analysed by western blot, respectively. B) Relative levels of IE proteins in (A) were quantified using Image J freeware after normalisation to actin levels. (TIF) [file pone.0036057.s002.tif]

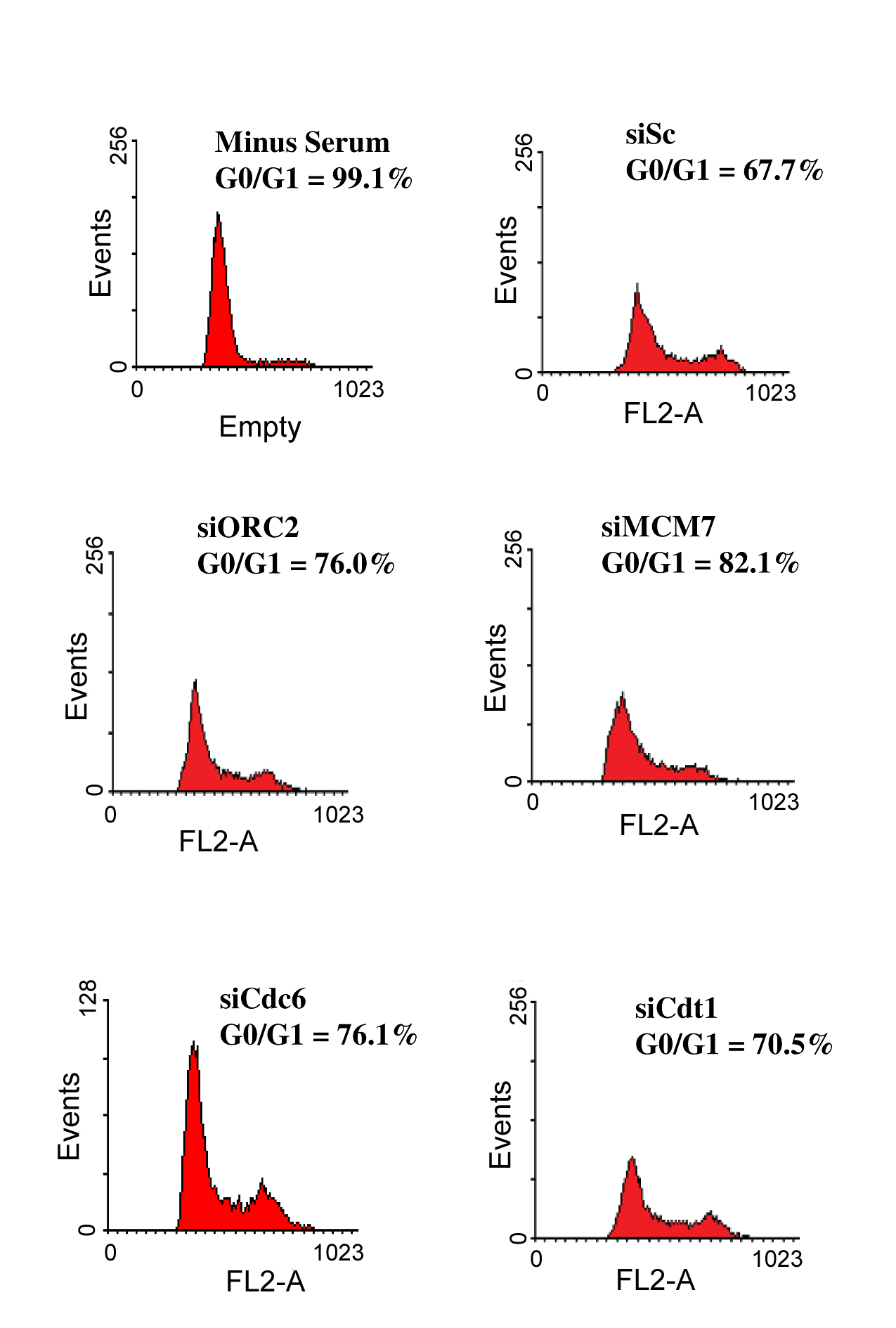

Supplement: Figure S3 — Effects of knockdown of ORC2, MCM7, Cdt1, and Cdc6 on cell cycle. HFFFs were transfected with siRNAs as labelled. After this, cells were cultured in EMEM-10 for 48 h and then stained with PI and cell cycle analysis was assayed by flow cytometry after gating for single cells. Percentages of cells in the G0/G1 phases of the cell cycle were calculated using winMDi software. Untreated cells in the absence of serum (minus serum) are also shown for comparison. (TIF) [file pone.0036057.s003.tif]
